# Estimating Dementia from Self-Reports of Diagnoses Among Adults Aged 65 and Over: United States, 2019

**DOI:** 10.1093/geroni/igab046.2764

**Published:** 2021-12-17

**Authors:** Ellen Kramarow

**Affiliations:** National Center for Health Statistics, Hyattsville, Maryland, United States

## Abstract

Prior research shows that, overall, about 10% of the population aged 65 and over in the U.S. has dementia. Estimating the prevalence of dementia from nationally representative surveys can be accomplished by asking respondents about a diagnosis, by administering a cognitive assessment, or, if available, by examining linked medical claims data. In 2019 for the first time, the National Health Interview Survey (NHIS) added “dementia, including Alzheimer’s disease” to the questions asking about doctor-diagnosed health conditions. Although estimates derived from doctor-diagnosed questions usually underestimate the true prevalence of a condition, and estimating dementia from self-reports presents additional challenges, they are still useful for many surveillance and research objectives. Early diagnosis of dementia is encouraged to allow patients and their families to plan for future needs. The objective of this research is to describe the noninstitutionalized population aged 65 and over who have a dementia diagnosis, by selected socio-demographic, health, and healthcare utilization characteristics. Point estimates, standard errors, and 95% confidence intervals for percentages are calculated using NHIS adult sample weights and adjusted for the complex sample design of NHIS. Preliminary analyses show that, overall, 4% of the 65 and over noninstitutionalized population has a diagnosis of dementia. About 8% of respondents with less than a high school education reported a dementia diagnosis compared with 2% of those with more than a high school education. Those with a dementia diagnosis were more likely to report depression than those without a dementia diagnosis (44% vs. 14%).

